# Clinical Response to Immunotherapy Targeting Programmed Cell Death Receptor 1/Programmed Cell Death Ligand 1 in Patients With Treatment-Resistant Microsatellite Stable Colorectal Cancer With and Without Liver Metastases

**DOI:** 10.1001/jamanetworkopen.2021.18416

**Published:** 2021-08-09

**Authors:** Chongkai Wang, Jaideep Sandhu, Ching Ouyang, Jian Ye, Peter P. Lee, Marwan Fakih

**Affiliations:** 1 Department of Medical Oncology and Therapeutics Research, City of Hope Comprehensive Cancer Center, Duarte, California; 2 Center for Informatics, City of Hope National Medical Center, Duarte, California; 3 Department of Computational and Quantitative Medicine, Beckman Research Institute of the City of Hope, Duarte, California; 4 Department of Immuno-oncology, Beckman Research Institute of the City of Hope, Duarte, California

## Abstract

**Importance:**

Microsatellite stable (MSS) metastatic colorectal cancer has been historically characterized as resistant to immunotherapy. Recent studies have demonstrated limited clinical activity of programmed cell death receptor 1/programmed death ligand 1 (PD-1/PD-L1) targeting in MSS metastatic colorectal cancer. The association of metastatic disease in the liver with treatment response has not been fully investigated.

**Objective:**

To investigate the association of liver metastases with response to PD-1/PD-L1–targeting therapy in MSS metastatic colorectal cancer.

**Design, Setting, and Participants:**

This single-center retrospective cohort study evaluated clinical responses to PD-1– or PD-L1–targeting therapy, with or without other investigational agents, in patients with MSS metastatic colorectal cancer and disease progression after standard of care therapy from January 1, 2014, to December 31, 2020.

**Main Outcomes and Measures:**

Objective response rate (ORR) and progression-free survival (PFS), measured from initiation of PD-1/PD-L1–targeting therapy.

**Results:**

Ninety-five patients with MSS metastatic colorectal cancer were identified (54 men [56.8%]; median age, 55 [interquartile range (IQR), 49-64] years). The overall ORR was 8.4% (8 of 95 patients). Eight of 41 patients without liver metastases achieved an ORR of 19.5%, and no response was observed in 54 patients with liver metastases. The disease control rate was 58.5% (24 of 41) in patients without liver metastasis and 1.9% (1 of 54) in patients with liver metastasis. Patients without liver metastases at the time of PD-1/PD-L1–targeting treatment had a superior median PFS compared with patients with liver metastases (4.0 [IQR, 2.0-7.5] vs 1.5 [IQR, 1.0-2.0] months; *P* < .001). In addition, median PFS was 5.5 (IQR, 2.0-11.5) months for patients without any prior or current liver involvement at the time of PD-1/PD-L1–targeting treatment initiation. Using a multivariate Cox regression model correcting for Eastern Cooperative Oncology Group status, primary tumor location, *RAS* and *BRAF* status, tumor mutation burden, and metastatic sites, liver metastases was the variable with the most significant association with faster progression after PD-1/PD-L1 treatment inhibition (hazard ratio, 7.00; 95% CI, 3.18-15.42; *P* < .001).

**Conclusions and Relevance:**

Findings of this cohort study suggest that patients with MSS metastatic colorectal cancer and without liver metastases may derive clinical benefits from checkpoint inhibitors, whereas the presence of liver metastases was associated with resistance. Further prospective studies are needed to investigate PD-1/PD-L1 inhibitors in patients with MSS metastatic colorectal cancer without liver metastases.

## Key Points

**Question:**

Is immunotherapy targeting programmed cell death receptor 1/programmed cell death ligand 1 (PD-1/PD-L1) associated with the presence of liver metastasis at the time of therapy and outcomes among patients with treatment-resistant microsatellite stable (MSS) metastatic colorectal cancer?

**Findings:**

This cohort study included 95 patients with MSS metastatic colorectal cancer. Patients without liver metastases had a significantly superior objective response rate (19.5% vs 0) and median progression-free survival (4.0 vs 1.5 months) compared with patients with liver metastases; multivariate analysis revealed that the presence of liver metastases was an independent prognostic factor associated with poor outcome of PD-1/PD-L1 therapy.

**Meaning:**

This cohort study suggests that PD-1/PD-L1 inhibitors should be reinvestigated in prospective trials in patients with MSS metastatic colorectal cancer without liver involvement.

## Teaser

This cohort study analyzes the association of metastatic disease pattern with response to programmed cell death receptor 1/programmed cell death ligand 1–targeting therapy in patients with treatment-resistant microsatellite stable metastatic colorectal cancer.

## Introduction

Microsatellite stable (MSS) colorectal cancers represent 95% of all metastatic colorectal cancer cases and are characterized by low tumor mutation burden (TMB) and low immune infiltration compared with microsatellite instability–high (MSI-H) colorectal tumors.^1^ As a result, programmed cell death receptor 1/programmed cell death ligand 1 (PD-1/PD-L1) inhibition has led to robust clinical benefit in MSI-H colorectal cancers, whereas limited antitumor activity has been observed in colorectal cancers with MSS.^2,3,4,5^

The Japanese REGONIVO (Regorafenib Plus Nivolumab in Patients With Advanced Gastric or Colorectal Cancer) trial^6^ reported a response rate of 33% and a median progression-free survival (PFS) of 7.9 months in 24 patients with MSS metastatic colorectal cancer who had disease progression with standard chemotherapy. In that study, patients with liver metastases had a response rate of 8.3% (1 of 12), whereas patients with lung metastases without liver involvement had a response rate of 63.6% (7 of 11).^6^ In a retrospective trial of refractory MSS colorectal cancer treated with regorafenib and nivolumab, Wang et al^7^ described a disease control rate (DCR) of 27.8% (5 with stable disease) in 18 patients, whereas no patients experienced an objective response. Stable disease occurred in patients without liver metastases at the time of enrollment.^7^ Similarly, the combination of regorafenib plus toripalimab (a PD-1 inhibitor) led to a response rate of 30.0% in patients with MSS colorectal cancer without liver metastases, whereas patients with liver metastases had an inferior response rate of 8.7%.^7^ More recently, the combination of avelumab and regorafenib was investigated in MSS colorectal cancer, with a reported DCR of 53.5%, whereas no objective responses were noted.^8^ These data, in summation, suggest a potential clinical benefit to regorafenib plus a PD-1– or PD-L1–targeting agent and suggest a preferential response in patients without hepatic metastatic disease.

Studies reporting on PD-1/PD-L1–targeting therapy in metastatic colorectal cancer in the context of sites of metastases have been limited by their small sample sizes and lack of multivariate analysis that integrates sidedness, results of molecular analysis, and TMB. To further investigate the association of metastatic disease pattern with response to PD-1– or PD-L1–targeting therapy in MSS colorectal cancer, we performed a single-center retrospective analysis of all patients with treatment-resistant MSS metastatic colorectal cancer treated at City of Hope Comprehensive Cancer Center, Duarte, California, with PD-1 or PD-L1 inhibitors during the last 6 years.

## Methods

### Patient Population

In this cohort study, we retrospectively reviewed the medical records of patients with metastatic colorectal cancer treated at City of Hope from January 1, 2014, to December 31, 2020. This cohort study was approved by the institutional review board of the City of Hope and followed the Strengthening the Reporting of Observational Studies in Epidemiology (STROBE) reporting guideline. Because this study was an outcome clinical trial, the institutional review board did not require informed consent.

Patients with MSS metastatic colorectal cancer who received PD-1/PD-L1–targeting therapy after progression with prior standard chemotherapy were eligible for study inclusion. Patients who received concomitant chemotherapy with PD-1 or PD-L1 therapy were excluded to avoid possible confounding. However, patients receiving additional investigational agents in addition to PD-1– or PD-L1–targeting therapy were allowed. In addition, patients receiving radiotherapy to 1 lesion or organ for the purpose of inducing an abscopal response were included. The identified patients were included regardless of whether they received treatment on an investigational trial or compassionate basis. Patient demographics and molecular characteristics, when available, were collected for all patients. Responses to treatment were assessed radiographically by Response Evaluation Criteria in Solid Tumors, version 1.1, guidelines. Disease progression was observed in 89 patients. Six patients were still receiving treatment with ongoing clinical benefit at the time of data analysis. Progression-free survival was defined as the interval from the dates of treatment initiation to disease progression.

### Statistical Analysis

Clinical characteristics were compared using the Wilcoxon rank sum test for age and TMB; categorical variables, using the Fisher exact test. We computed 95% CIs using the Wald confidence limits for the binomial proportion. Cox proportional hazard regression was used to determine the association of demographic and clinical variables with response and PFS. The full model included terms for primary tumor site, metastatic location, age, sex, and *RAS* (including *KRAS* [OMIM 190070] and *NRAS* [OMIM 164790]/*BRAF* [OMIM 164757]) status. Progression-free survival curves were constructed with the Kaplan-Meier method. Analyses were performed using R, version 4.0.3 (R Program for Statistical Computing). All tests were 2 sided, with *P* < .05 considered statistically significant.

## Results

### Baseline Patient Population Characteristics

Ninety-five patients with MSS metastatic colorectal cancer met the eligibility criteria (41 women [43.2%] and 54 men [56.8%]; median age, 55 [interquartile range (IQR), 49-64] years). Baseline patient demographics and molecular tumor characteristics are detailed in Table 1. Metastatic disease was most prevalent in the lungs (66 patients [69.5%]) and liver (54 patients [56.8%]). Peritoneal metastases were found in 29 patients (30.5%); distant lymph node metastases in 50 patients (52.6%); brain metastases in 3 patients (3.2%); and bone metastases in 10 patients (10.5%).

**Table 1.** Baseline Characteristics of Patients and Corresponding ORRs and DCRs

Characteristic	Patient group^a^					ORR, %^b^	*P* value^c^	DCR, %^d^	*P* value^c^
	All (N = 95)	Best overall response							
		Complete (n = 1)	Partial (n = 7)	Stable disease (n = 17)	Progressive disease (n = 70)				
Age, median (IQR), y	55 (49-64)	47 (47-47)	62 (50-63)	55 (47-57)	55 (49-66)	NA	.86	NA	.27
Sex									
Female	41 (43.2)	0	6 (85.7)	6 (35.3)	29 (41.4)	14.6	.07	29.3	.64
Male	54 (56.8)	1 (100)	1 (14.3)	11 (64.7)	41 (58.6)	3.7		24.1	
Primary tumor									
Left-sided	69 (72.6)	1 (100)	3 (42.9)	16 (94.1)	49 (70.0)	5.8	.21	29.0	.44
Right-sided	26 (27.4)	0	4 (57.1)	1 (5.9)	21 (30.0)	15.4		19.2	
ECOG status									
0	42 (44.2)	1 (100)	6 (85.7)	10 (58.8)	25 (35.7)	16.7	.02	40.5	.01
1	53 (55.8)	0	1 (14.3)	7 (41.2)	45 (64.3)	1.9		15.1	
Metastasis site^e^									
Peritoneal									
Involved	29 (30.5)	0	1 (14.3)	5 (29.4)	23 (32.9)	3.4	.43	20.7	.46
Noninvolved	66 (69.5)	1 (100)	6 (85.7)	12 (70.6)	47 (67.1)	0.6		28.8	
Bone									
Involved	10 (10.5)	0	0	2 (11.8)	8 (11.4)	0	.59	20.0	>.99
Noninvolved	85 (89.5)	1 (100)	7 (100)	15 (88.2)	62 (88.6)	9.4		27.1	
Brain									
Involved	3 (3.2)	0	0	1 (5.9)	2 (2.9)	0	>.99	33.3	>.99
Noninvolved	92 (96.8)	1 (100)	7 (100)	16 (94.1)	68 (97.1)	8.7		26.1	
Liver									
Involved	54 (56.8)	0	0	1 (5.9)	53 (75.7)	0	<.001	1.9	<.001
Noninvolved	41 (43.2)	1 (100)	7 (100)	16 (94.1)	17 (24.3)	19.5		58.5	
Lung									
Involved	66 (69.5)	0	3 (42.9)	13 (76.5)	50 (71.4)	4.5	.054	24.2	.61
Noninvolved	29 (30.5)	1 (100)	4 (57.1)	4 (23.5)	20 (28.6)	17.2		31.0	
Lymph node									
Involved	50 (52.6)	1 (100)	5 (71.4)	7 (41.2)	37 (52.9)	12.0	.27	26.0	>.99
Noninvolved	45 (47.4)	0	2 (28.6)	10 (58.8)	33 (47.1)	4.4		26.7	
Variant status									
*APC* ^f^									
Altered	50 (71.4)	1 (100)	3 (60.0)	13 (81.3)	33 (68.8)	8.0	>.99	34.0	.57
Nonaltered	20 (28.6)	0	2 (40.0)	3 (18.7)	15 (31.3)	10.0		25.0	
*BRAF* V600E									
Altered	4 (4.2)	0	2 (28.6)	0	2 (2.9)	50.0	.03	50.0	.28
Nonaltered	91 (95.8)	1 (100)	5 (71.4)	17 (100)	68 (97.1)	6.5		25.3	
*RAS* ^g^									
Altered	58 (61.1)	0	2 (28.6)	10 (58.8)	46 (65.7)	3.4	.053	20.7	.15
Nonaltered	37 (38.9)	1 (100)	5 (71.4)	7 (41.2)	24 (34.3)	16.2		35.1	
*TP53* ^h^									
Altered	56 (78.9)	1 (100)	5 (100)	12 (75.0)	38 (77.6)	10.7	.33	32.1	.76
Nonaltered	15 (21.1)	0	0	4 (25.0)	11 (22.4)	0		26.7	
TMB, median (IQR)^i^	5.0 (3-7)	3 (3-3)	4 (2-10)	5 (4-7)	4 (3-7)	NA	.65	NA	.61

Abbreviations: DCR, disease control rate; ECOG, Eastern Cooperative Oncology Group; IQR, interquartile range; NA, not applicable; ORR, objective response rate; TMB, tumor mutation burden.

^a^ Unless otherwise indicated, data are expressed as number (%) of patients. Percentages have been rounded and may not total 100. All percentages are based on weighted analysis.

^b^ Calculated as complete plus partial responses vs stable plus progressive disease.

^c^ Calculated using the Fisher exact test except for age and TMB (Wilcoxon rank sum test).

^d^ Calculated as complete plus partial responses plus stable disease vs progressive disease.

^e^ Indicates at treatment.

^f^ Data were missing for 25 cases.

^g^ Including *KRAS* and *NRAS*.

^h^ Data were missing for 24 cases.

^i^ Data were missing for 32 cases.

All patients had received at least 2 lines of prior systemic therapy, including anti–epidermal growth factor receptor if left-sided and *RAS/BRAF* wild-type. Anti–PD-1 and anti–PD-L1 therapy consisted of nivolumab (55 patients), atezolizumab (17 patients), pembrolizumab (13 patients), and durvalumab (10 patients). Concurrent therapy with PD-1 or PD-L1 inhibitors was divided into 5 categories: vascular endothelial growth factor receptor (VEGFR) inhibitors (45 patients), mitogen-activated protein kinase inhibitors (6 patients), cytotoxic T-lymphocyte–associated protein 4 (CTLA-4) inhibitors (9 patients), radiotherapy (9 patients), and several other targeted or biological agents (17 patients).

### Patient Characteristics and Treatment Response to PD-1 or PD-L1 Inhibitors

All patients were evaluable for best response. We observed an overall objective response rate (ORR) in 8 of 95 patients (8.4%; 95% CI, 3.7%-15.9%) in our analysis, with 1 complete radiographic response and 7 partial responses. In addition, 17 patients (17.9%) had stable disease and 70 (73.7%) had progressive disease as a best response (Table 1). Univariate analysis revealed an Eastern Cooperative Oncology Group (ECOG) status of 0 (7 of 42 [16.7%]; *P* = .02) and *BRAF *V600E alteration (2 of 4 [50.0%]; *P* = .03) were associated with better ORR. No objective response was observed in patients with liver metastases. In contrast, responses were noted in 8 of 41 patients without liver metastasis (19.5%; 1 complete response and 7 partial responses; *P* < .001) (Table 1). The ORR was 4.5% in patients with lung metastases (*P* = .05) and 12.0% in patients with lymph node metastases (*P* = .27). The DCR was significantly higher in patients without liver metastases (24 of 41 [58.5%]) in comparison with patients with liver metastases (1 of 54 [1.9%]; *P* < .001). In addition, an ECOG performance status of 0 was also associated with a higher DCR (17 of 42 [40.5%] vs 8 of 53 [15.1%]; *P* = .01) when compared with an ECOG performance status of 1 (Table 1).

### Patient Characteristics and PFS With PD-1 or PD-L1 Inhibitors

**Figure 1.**
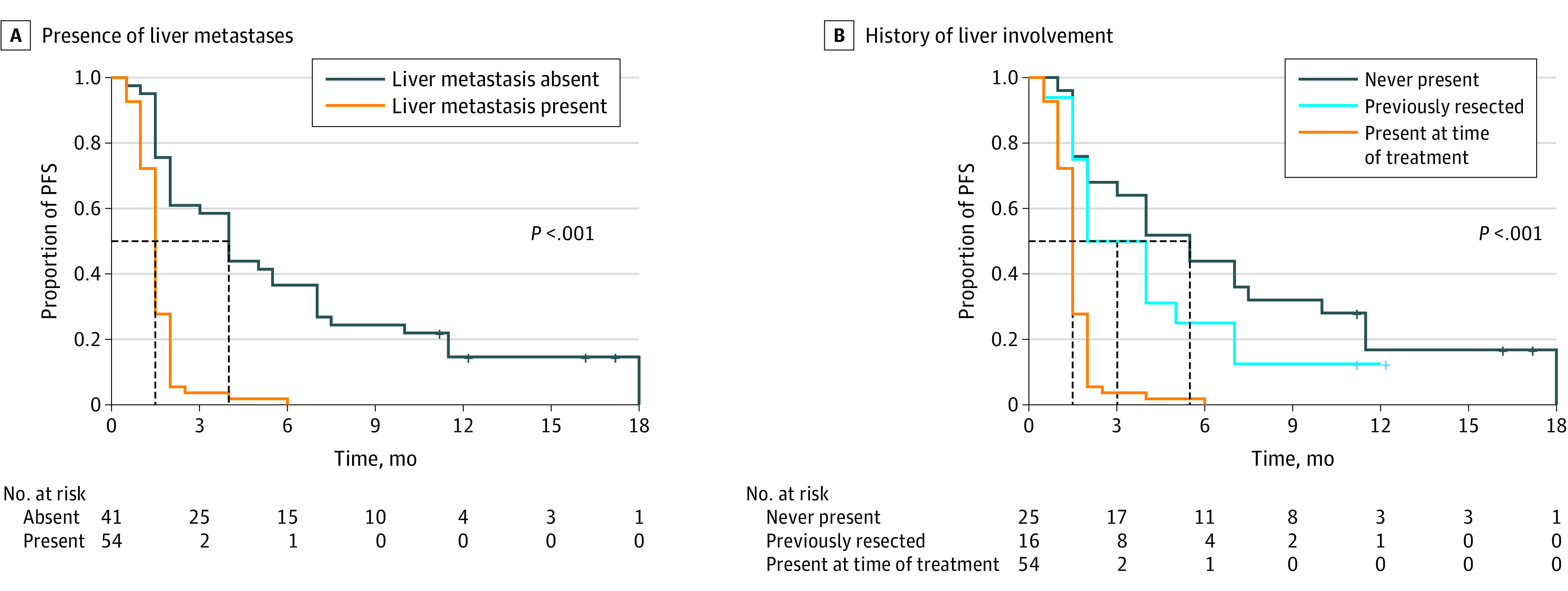
Kaplan-Meier Curves for Progression-Free Survival (PFS) Among Patients With Microsatellite Stable Metastatic Colorectal Cancer A, Kaplan-Meier curves for PFS stratified by presence (n = 54) or absence (n = 41) of liver metastasis at the time of enrollment. B, Kaplan-Meier curves for PFS stratified by patients who never had liver involvement, had prior liver resection but no liver involvement at the time of treatment initiation, and had liver metastases at the time of treatment initiation.

**Figure 2.**
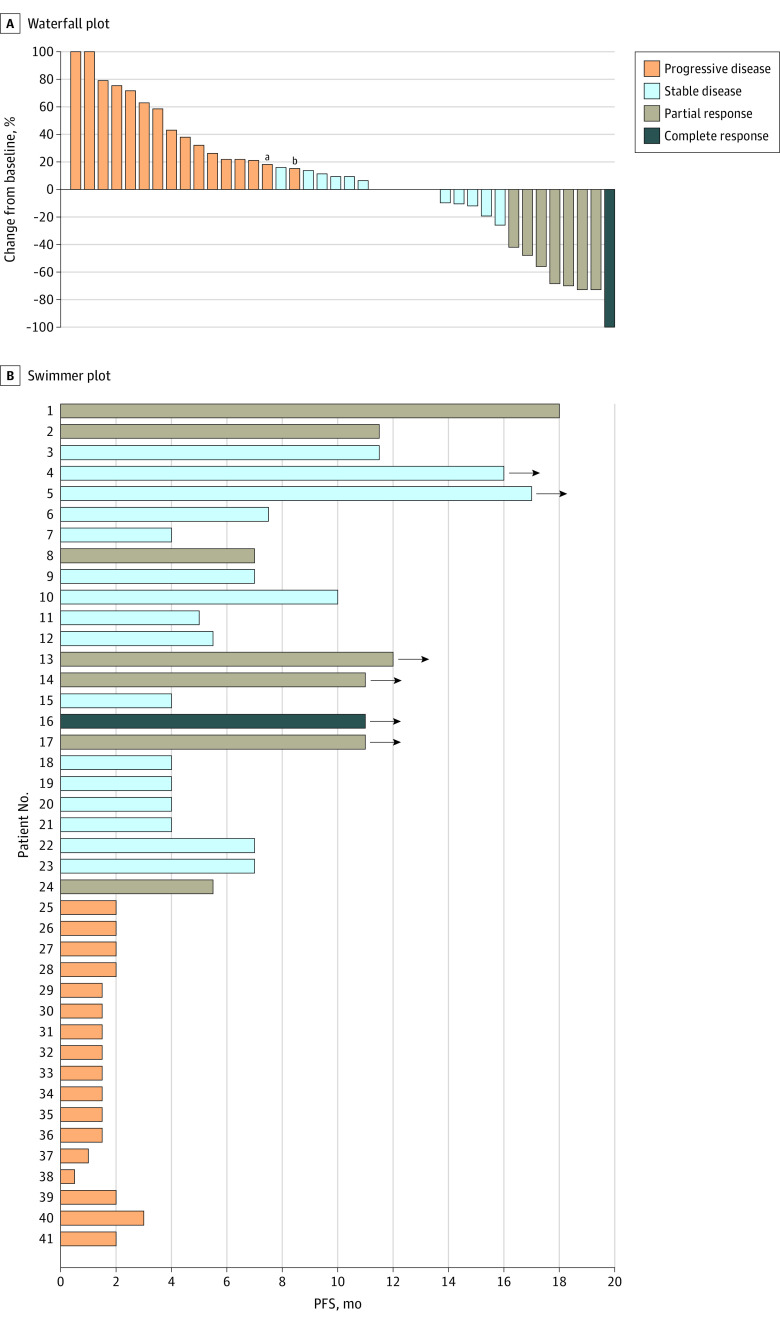
Tumor Response in Patients With Microsatellite Stable Metastatic Colorectal Cancer Without Liver Metastases A, Waterfall plot of tumor response in patients without liver metastases. B, Swimmer plot presentation of progression-free survival (PFS) in patients without liver metastases. Arrow indicates ongoing benefit. ^a^Indicates new lesion. ^b^Indicates clinical progression.

**Figure 3.**
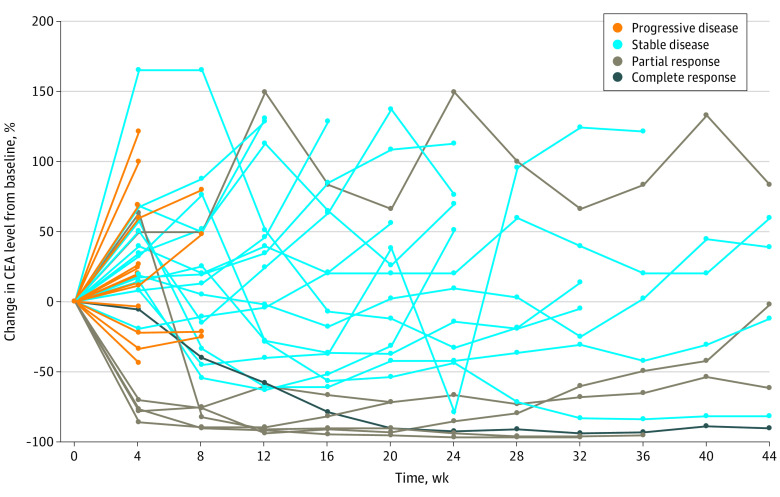
Carcinoembryonic Antigen (CEA) Response in Patients With Microsatellite Stable Metastatic Colorectal Cancer Without Liver Metastases Spider plot shows change in the CEA level from baseline.

Kaplan-Meier analysis demonstrated a median PFS of 4.0 (IQR, 2.0-7.5) months for patients without liver metastases vs 1.5 (IQR, 1.0-2.0) months for patients with liver metastases (*P* < .001). The 6-month PFS rate was 36.6% in patients without vs 1.9% in patients with liver metastases (Figure 1A). Univariate analysis showed that the presence of liver metastases at the time of treatment initiation was significantly associated with shorter PFS (hazard ratio [HR], 4.98; 95% CI, 2.95-8.38; *P* < .001). None of the other variables were associated with PFS outcome (Table 2). A multivariate model including age, sex, primary tumor location, ECOG status, metastatic site, TMB, and *APC* (OMIM 611731), *RAS*, *BRAF* V600E, and *TP53* (OMIM 191170) alterations maintained that liver metastases at the time of treatment initiation was the most significant factor associated with worse PFS (HR, 7.00; 95% CI, 3.18-15.42; *P* < .001). In addition, *RAS* mutation (HR, 2.78; 95% CI, 1.19-6.47) and right-sided tumors (HR, 2.34; 95% CI, 1.07-5.13) were associated with worse PFS on multivariate analysis, but the statistical significance was marginal (Table 2). We further explored the cohort of patients without liver metastasis at enrollment in 2 groups: patients without any history of liver involvement (n = 25) and patients with a history of liver lesion resection but without active liver disease at the time of treatment (n = 16). Kaplan-Meier analysis showed that the median PFS for patients without any history of liver involvement was 5.5 (IQR, 2.0-11.5) months vs 3.0 (IQR, 1.8-6.0) months for patients with history of liver resection. The 6-month PFS rate was 44.0% (11 of 25) vs 25.0% (4 of 16) for patients without and with any history of liver involvement, respectively. Patients with liver metastases at the time of treatment had significantly inferior median PFS than patients with history of liver metastasectomy but without active liver disease at the time of treatment (1.5 [IQR, 1.0-2.0] vs 3.0 [IQR, 1.8-6.0] months; HR, 2.37; 95% CI, 1.45-3.86; *P* < .001) (Figure 1B). To further demonstrate the clinical benefit of PD-1/PD-L1 inhibition in patients with MSS colorectal cancer without liver metastases, we performed a waterfall and swimmer plot data analysis. As shown in Figure 2, significant tumor shrinkage and durable tumor responses (PFS, >6 months) were recorded in 13 of 41 patients (31.7%) and 15 of 41 patients (36.6%), respectively, in this population. A few patients achieved a PFS of 12 months, and the benefit was still ongoing. In addition, significant and protracted declines in levels of carcinoembryonic antigen (a decline of greater than 70% was observed in 5 responding patients for at least 36 weeks) were also recorded in patients without liver metastasis (Figure 3).

**Table 2.** PFS and Association With Clinicopathologic Characteristics Using Cox Regression

Clinicopathologic variable	Univariate analysis		Multivariate analysis	
	HR (95% CI)	*P* value	HR (95% CI)	*P* value
Age at diagnosis				
Continuous	1.00 (0.98-1.02)	.73	NA	NA
≥65 vs <65 y	1.31 (0.81-2.13)	.27	1.87 (0.93-3.76)	.08
Female vs male	0.91 (0.60-1.40)	.68	1.13 (0.55-2.34)	.73
Right- vs left-sided primary tumor	1.21 (0.76-1.95)	.42	2.34 (1.07-5.13)	.03
ECOG status 1 vs 0	1.43 (0.93-2.19)	.11	0.78 (0.37-1.65)	.52
Metastasis site, involved vs noninvolved^a^				
Peritoneal	1.58 (1.00-2.48)	.049	1.18 (0.54-2.60)	.68
Liver	4.98 (2.95-8.38)	<.001	7.00 (3.18-15.42)	<.001
Lung	1.04 (0.66-1.64)	.88	0.94 (0.40-2.21)	.89
Lymph	0.97 (0.63-1.47)	.88	1.02 (0.50-2.09)	.95
Bone	1.09 (0.54-2.17)	.81	0.58 (0.21-1.59)	.29
Brain	0.89 (0.28-2.82)	.84	1.95 (0.33-11.62)	.46
Alteration vs no alteration				
*APC* ^b^	0.70 (0.41-1.21)	.20	0.59 (0.27-1.28)	.18
*RAS* ^c^	1.47 (0.95-2.29)	.08	2.78 (1.19-6.47)	.02
*BRAF* V600	0.48 (0.15-1.54)	.22	0.22 (0.04-1.31)	.10
*TP53* ^d^	0.91 (0.51-1.66)	.77	2.10 (0.84-5.24)	.11
TMB (continuous)^e^	0.99 (0.93-1.06)	.84	0.98 (0.88-1.08)	.63

Abbreviations: ECOG, Eastern Cooperative Oncology Group; HR, hazard ratio; NA, not applicable; PFS, progression-free survival; TMB, tumor mutational burden.

^a^ Indicates at treatment.

^b^ Data were missing for 25 cases.

^c^ Includes *KRAS* and *NRAS*.

^d^ Data were missing for 24 cases.

^e^ Data were missing for 32 cases.

## Discussion

Colorectal cancer with MSS has long been considered resistant to PD-1/PD-L1 blockade.^2,3,4,5^ However, the promising response rate of 33% seen in the Japanese REGONIVO trial^6^ has revitalized interest in immunotherapy in MSS colorectal cancer. In that small study, patients with liver metastases had an inferior response rate compared with patients with lung metastases (8.3% vs 50.0%).^6^ Four other independent early trials^7,8,9,10^ have also demonstrated promising clinical activity of various anti-VEGFR agents plus PD-1/PD-L1 blockade in MSS colorectal cancer, but the responses were considerably lower than those in the REGONIVO trial. One of those studies^7^ correlated the response to immunotherapy with the metastatic disease pattern and showed that patients without liver metastases had response rates superior to those of patients with liver metastases (30.0% vs 8.7%). In addition, similar findings of relative clinical resistance to PD-1/PD-L1–targeting agents were noted in patients with liver metastases in melanoma, lung cancer, kidney cancer, and other tumor types.^11,12,13,14,15,16^ Two recent retrospective studies^17,18^ including more than 300 patients with solid tumors who received checkpoint blockade demonstrated that the presence of liver metastases was associated with worse PFS and overall survival regardless of tumor histology. Our study demonstrates that PD-1/PD-L1 inhibition provides significant clinical benefit to a group of patients with MSS colorectal cancer without liver metastases, whereas the presence of liver metastases correlates with lack of response.

In melanoma and non–small cell lung cancer, the presence of liver metastases was not only associated with decreased activation of tumor-infiltrating CD8^+^ T cells, but also with decreased CD8^+^ T-cell infiltration in primary tumors.^19,20^ In addition, lower CD8^+^ T-cell infiltration was also found in extrahepatic metastatic lesions from patients with liver metastases compared with patients without liver metastases.^11^ These findings suggest liver metastases may induce a systemic immunosuppressive effect, thereby inhibiting antitumor immunity, and undermine the efficacy of checkpoint inhibitors in this population. A recent preclinical study using a dual-tumor immunocompetent mouse model demonstrated that concurrent tumor inoculation in the liver significantly suppressed systemic anti–tumor immune response via regulatory T cell–modulated myeloid-derived suppressor cell immune suppression. Although PD-1 inhibition alone showed limited antitumor response in this preclinical model, combining regulatory T cell–depleting agents such as CTLA-4 inhibitor with PD-1 inhibition successfully reversed the suppressive immune state induced by liver metastases.^21^ Another recent study^22^ also revealed that liver metastases limit the efficacy of immunotherapy systemically in the clinical and preclinical settings. In multiple preclinical models, this study showed liver metastasis attracts immunosuppressive macrophages that induce apoptosis of tumor antigen–specific T cells within the liver. This leads to a loss of tumor-reactive T cells systemically and subsequently to diminished efficacy of immunotherapy.^22^ These studies support the notion that liver metastatic tumors not only suppress intrahepatic antitumor immune response, but also inhibit systemic antitumor immunity, which leads to diminished response to PD-1/PD-L1 inhibition in this population. The clinical benefits that we observed with PD-1/PD-L1 inhibition in patients with resected liver metastases support the above hypotheses. However, the clinical relevance of CTLA-4 inhibition in addition to PD-1/PD-L1 inhibition remains to be demonstrated, especially in light of the limited efficacy of durvalumab and tremelimumab in MSS metastatic colorectal cancer.^23,24^

In our patients without liver metastases, responses to checkpoint blockade were mostly seen in patients with lung and lymph node metastases (eTable 1 in the Supplement). Patients with peritoneal metastases without liver metastases had longer PFS than patients with liver metastases (3.0 vs 1.5 months) but appeared to have a worse outcome than patients without liver and peritoneal disease (eFigure 1 in the Supplement). In our population, responses to anti–PD-1/PD-L1 agents were not associated with any investigational partner associated with checkpoint inhibitors. The benefits occurred irrespective of the use or lack of use of concurrent VEGFR inhibitors, such as regorafenib. In patients who received a VEGFR inhibitor plus PD-1 inhibitor, the ORR was 15.4% in patients without liver metastases compared with 0 in patients with liver metastases. The median PFS was 4.0 months in patients without liver metastases vs 1.5 months in patients with liver metastases (eTable 2 and eFigure 2 in the Supplement). Similarly, we did not see any association between benefit or lack of benefit in ORR with the addition of CTLA-4 therapy, radiotherapy, or mitogen-activated protein kinase inhibitor therapy (eTable 2 in the Supplement). The DCRs were similar across all 5 different subgroups of PD-1/PD-L1–based therapy, even when stratified by liver and nonliver metastases (eTable 2 in the Supplement). However, given the small size of some of the subgroups and the retrospective nature of our study, definitive conclusions on the effect of additional therapies such as CTLA-4 inhibition or radiotherapy cannot be made. The benefits seen in the nonliver metastases group cannot be attributed solely to the better prognosis of patients with nonhepatic metastatic disease, particularly because they pertain to lung metastases only.^25,26^ The significant tumor shrinkage seen in our waterfall plot analysis and the protracted PFS shown in the swimmer plot, with 36.6% PFS at 6 months in patients with nonliver metastases, indicates an association with treatment in this population.

The significant clinical benefit seen in patients with history of liver resection in our study suggests that the systemic immune suppression of liver metastasis may be partially alleviated by resection or ablation. Although patients with liver metastases only achieve long-term survival with surgery, as many as 70% of these patients experience extrahepatic relapse.^27,28,29,30^ These patients can derive a benefit from PD-1/PD-L1–targeting therapy and thus should be included in future studies incorporating these agents.

Our study demonstrates that *RAS* variants were associated with poor outcome of checkpoint inhibition on multivariate analysis. Recent preclinical and clinical studies^31^ have demonstrated that oncogenic *RAS* alteration leads to an immune suppressive tumor microenvironment in MSS colorectal cancer by suppressing the expression of interferon-responsive genes and upregulating chemokine ligand 3, which attracts myeloid-derived suppressor cells to the tumor microenvironment and subsequently confers resistance to PD-1 inhibition. Interestingly, a diminished benefit with *RAS* alteration was recently noted with pembrolizumab in metastatic MSI-H colorectal cancer.^32^

High TMB is a predictive factor associated with response to checkpoint inhibition in MSI-H colorectal cancer.^33^ In addition, a recent prospective study of patients with advanced solid tumors^34^ demonstrated that TMB of greater than 10 was associated with increased response rate to pembrolizumab monotherapy regardless of microsatellite status. However, we did not see an association between TMB and response rate in our study. In the 7 patients who had an objective response, 2 tumors had a TMB of 10 to 20 (19 and 16), whereas the other 5 tumors had a TMB of 5 or less (eTable 1 in the Supplement). The presence of responses in patients with TMB of 5 or less is intriguing and suggests that additional factors beyond tumor mutation load dictate the potential benefit from PD-1/PD-L1 targeting.

Previous studies^35,36,37^ have shown that aberrant activation of WNT/β-catenin signaling was associated with immune exclusion in the tumor microenvironment and resistance to checkpoint blockade across human cancers. In our study, benefits to PD-1/PD-L1 targeting were seen in patients irrespective of the presence or lack of *APC* alteration, suggesting that WNT activation does not confer absolute clinical resistance to immunotherapy. We have noted a higher response rate in colorectal cancers with *BRAF* V600E alteration. Tumors with *BRAF* V600E alteration are enriched with consensus molecular subtype 1, which is characterized by rich immune infiltration in the tumor microenvironment.^38,39^ In our analysis, 2 of the 4 patients with *BRAF* V600E alteration had a partial response as the best response to PD-1/PD-L1 targeting. These results indicate *BRAF* V600E alteration is associated with a unique tumor immune microenvironment that may increase the chance of response to PD-1/PD-L1 inhibition. These findings need to be validated in a larger patient population set.

### Strengths and Limitations

To our knowledge, our study is (1) the largest to investigate the clinical response of anti–PD-1/PD-L1–targeting therapy in a population with refractory MSS colorectal cancer and (2) the first to investigate the association of liver metastases with response and PFS in a multivariable analysis that includes tumor genomics, TMB, and sidedness. Hepatic metastatic disease was the most significant variable associated with resistance to anti–PD-1/PD-L1 reagents. Our findings call for stratification by site of metastatic disease (liver vs no liver) in all future randomized clinical trials with anti–PD-1/PD-L1 reagents.

We acknowledge that our study has limitations. Given that it is a retrospective study, the results are inherently subject to patient selection bias. For example, our study was enriched by patients with nonliver metastatic disease. Our earlier observations of benefit in nonliver metastatic disease have led to a preferential extension of compassionate anti–PD-1 agents to this group of patients, therefore increasing their representation. However, the imbalance in liver and nonliver metastatic disease in our cohort should not diminish the clinical findings. Additional limitations include variations in anti–PD-1/PD-L1 agents and their combinations. The clinical benefits experienced from nivolumab, pembrolizumab, and atezolizumab in prior studies^2,3,4^ have been consistent and are not likely to alter our findings substantially. The inclusion of various investigational cotreatments was necessary to increase our patient pool. Tyrosine kinase inhibitors that target VEGFR (regorafenib and lenvatinib) have recently shown promise when combined with PD-1 targeting. Therefore, it is unsurprising that the combination of VEGFR plus PD-1 represented about half the patients on this study. Other combination therapies included combinations of PD-1/PD-L1 and CTLA-4 inhibitors with or without radiotherapy, and combinations of anti–PD-1/PD-L1 with a variety of other targeted agents. Those combinations represented small cohorts and did not justify us performing subgroup analyses. Irrespective of the subgroups of treatment, the benefits from checkpoint inhibition in our study were limited to nonliver metastases.

## Conclusions

The results of this cohort study demonstrate that the presence of liver metastases is associated with resistance to PD-1/PD-L1 inhibition in MSS metastatic colorectal cancer. Lack of liver metastases identifies a group of patients who may derive durable clinical benefits from PD-1/PD-L1 inhibition. Our findings support further investigation of PD-1/PD-L1 inhibitors in prospective trials in patients with MSS metastatic colorectal cancer without liver metastases. Additional preclinical and clinical efforts should be exerted to identify and overcome mechanisms of PD-1/PD-L1 resistance in colorectal cancer with liver metastases.

## Notes

Supplement. **eTable 1.** Characteristics of Patients With a Partial/Complete Response (PR/CR) as Best Response **eTable 2.** Responses by Treatment Reagents **eFigure 1.** Kaplan-Meier Curve for PFS in MSS Metastatic Colorectal Cancer by Metastatic Sites **eFigure 2.** Kaplan-Meier Curve for PFS in Patients Treated With VEGFR Inhibitor Plus PD-1 Inhibitor
