# Supplementary material for: Clinical Response to Immunotherapy Targeting Programmed Cell Death Receptor 1/Programmed Cell Death Ligand 1 in Patients With Treatment-Resistant Microsatellite Stable Colorectal Cancer With and Without Liver Metastases
Source: JAMA Netw Open. 2021 Aug 9;4(8):e2118416. doi: 10.1001/jamanetworkopen.2021.18416 (PMC8353537; doi:10.1001/jamanetworkopen.2021.18416)
Supplement: Supplement. — eTable 1. Characteristics of Patients With a Partial/Complete Response (PR/CR) as Best Response eTable 2. Responses by Treatment Reagents eFigure 1. Kaplan-Meier Curve for PFS in MSS Metastatic Colorectal Cancer by Metastatic Sites eFigure 2. Kaplan-Meier Curve for PFS in Patients Treated With VEGFR Inhibitor Plus PD-1 Inhibitor [file jamanetwopen-e2118416-s001.pdf]

Supplementary Online Content

Wang C, Sandhu J, Ouyang C, Ye J, Lee PP, Fakih M. Clinical response to immunotherapy targeting programmed cell death receptor 1/programmed cell death ligand 1 in patients with treatment-resistant microsatellite stable colorectal cancer with and without liver metastases. *JAMA Netw Open*. 2021;4(8):e2118416. doi:10.1001/jamanetworkopen.2021.18416

eTable 1. Characteristics of Patients With a Partial/Complete Response (PR/CR) as Best Response

eTable 2. Responses by Treatment Reagents

eFigure 1. Kaplan-Meier Curve for PFS in MSS Metastatic Colorectal Cancer by Metastatic Sites

eFigure 2. Kaplan-Meier Curve for PFS in Patients Treated With VEGFR Inhibitor Plus PD-1 Inhibitor

This supplementary material has been provided by the authors to give readers additional information about their work.

© 2021 Wang C et al. *JAMA Network Open*.

eTable 1. Characteristics of Patients With a Partial/Complete Response (PR/CR) as Best Response

Best Response	Age	Gender	Primary tumor location	ECOG status	Treatment	Liver	Lung	Lymph node	Peritoneal	*APC*	*RAS*	*BRAF*	*TP53*	TMB
PR	43	Female	Right	1	PD-1	No	Yes	Yes	No	MT	WT	V600E	MT	5
PR	63	Male	Left	0	PD-L1+CTLA-4	No	No	Yes	No	NA	MT	WT	NA	2
PR	62	Female	Right	0	PD-1+IDO1	No	No	Yes	No	WT	WT	V600E	MT	3
PR	49	Female	Left	0	VEGFR+PD-1	No	Yes	No	No	WT	WT	WT	MT	4
CR	47	Male	Left	0	VEGFR+PD-1	No	No	Yes	No	MT	WT	WT	MT	3
PR	63	Female	Right	0	VEGFR+PD-1	No	Yes	Yes	No	NA	MT	WT	NA	19
PR	69	Female	Right	0	PD-L1	No	No	No	Yes	MT	WT	WT	MT	16
PR	51	Female	Left	0	VEGFR+PD-1	No	No	Yes	No	MT	WT	WT	MT	2

CR: complete response; PR: partial response.

© 2021 Wang C et al. *JAMA Network Open*.

eTable 2. Responses by Treatment Reagents

	PD-1/PD-L1 monotherapy (n=18)		VEGFR inhibitor+anti-PD-1 (n=45)		RT+anti-PD-L1+anti-CTLA-4 (n=9)		MEK inhibitor+PD-1/PD-L1 inhibitor (n=6)		PD-1/PD-L1 inhibitor+ others* (n=17)	
ORR	11.1% (n=2)		8.9% (n=4)		11.1% (n=1)		0% (n=0)		5.9% (n=1)	
	Liver + (n=9)	Liver – (n=9)	Liver + (n=19)	Liver – (n=26)	Liver + (n=8)	Liver – (n=1)	Liver + (n=4)	Liver – (n=2)	Liver + (n=14)	Liver – (n=3)
CR	0	0	0	3.8% (1)	0	0	0	0	0	0
PR	0	22.2% (2)	0	11.5% (3)	0	1	0	0	0	33.3% (1)
SD	11.1% (1)	44.4% (4)	0	38.5% (10)	0	0	0	50% (1)	0	33.3% (1)
PD	88.9% (8)	33.3% (3)	100% (19)	46.2% (12)	100% (8)	0	100% (4)	50% (1)	100% (14)	33.3% (1)
DCR	11.1%	66.6%	0%	53.8%	0%	100%	0%	50%	0%	66.6%

RT: radiation therapy; ORR: objective response rate; CR: complete response; PR: partial response; SD: stable disease; PD: progressive disease; DCR: disease control rate

Liver +: with liver metastasis at the time of treatment

Liver -: without liver metastasis at the time of treatment

*Checkpoint inhibitors other than anti-PD-1/PD-L1 and anti-CTLA-4

© 2021 Wang C et al. *JAMA Network Open*.

eFigure 1. Kaplan-Meier Curve for PFS in MSS Metastatic Colorectal Cancer by Metastatic Sites

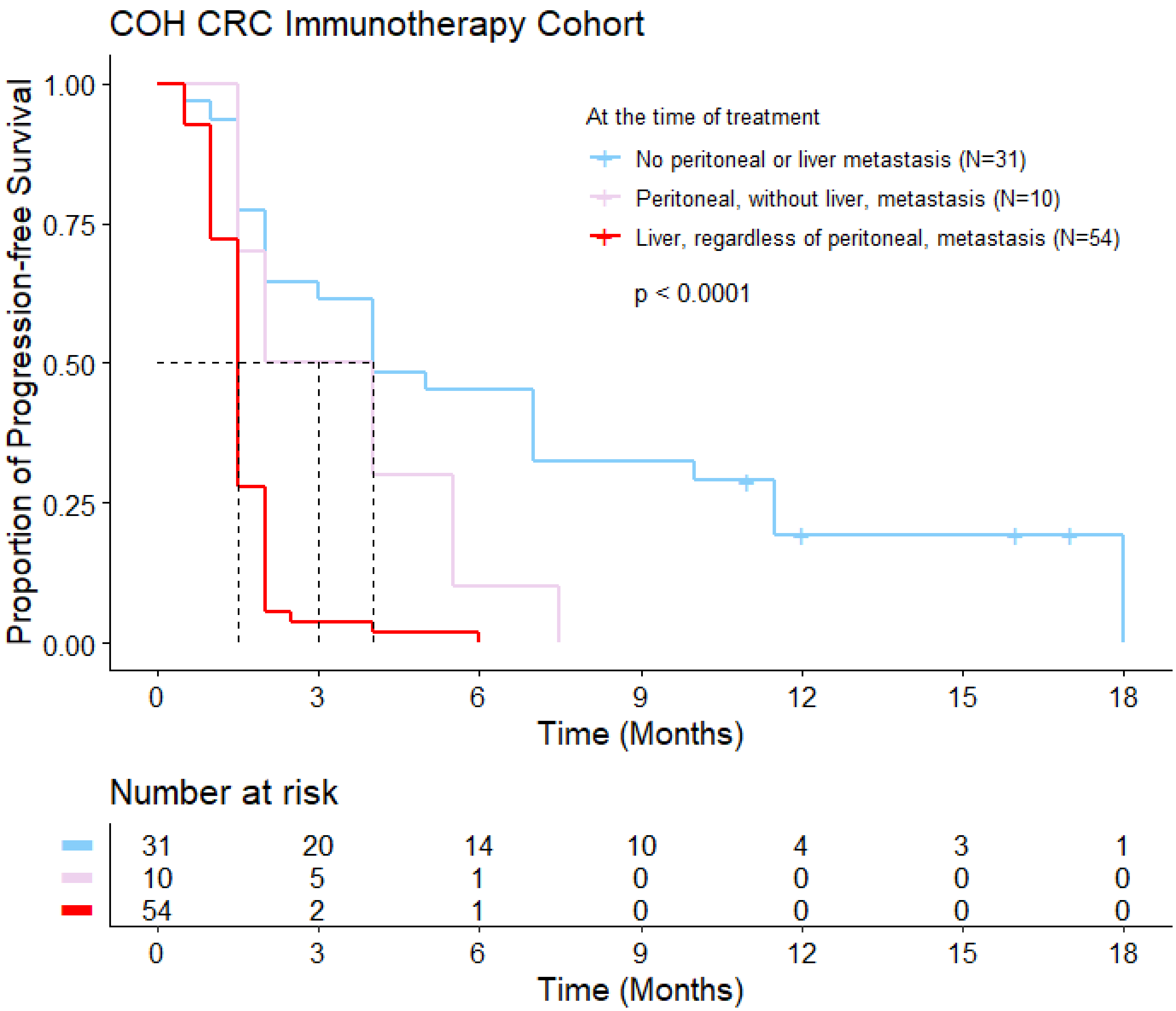

© 2021 Wang C et al. *JAMA Network Open*.

eFigure 2. Kaplan-Meier Curve for PFS in Patients Treated With VEGFR Inhibitor Plus PD-1 Inhibitor

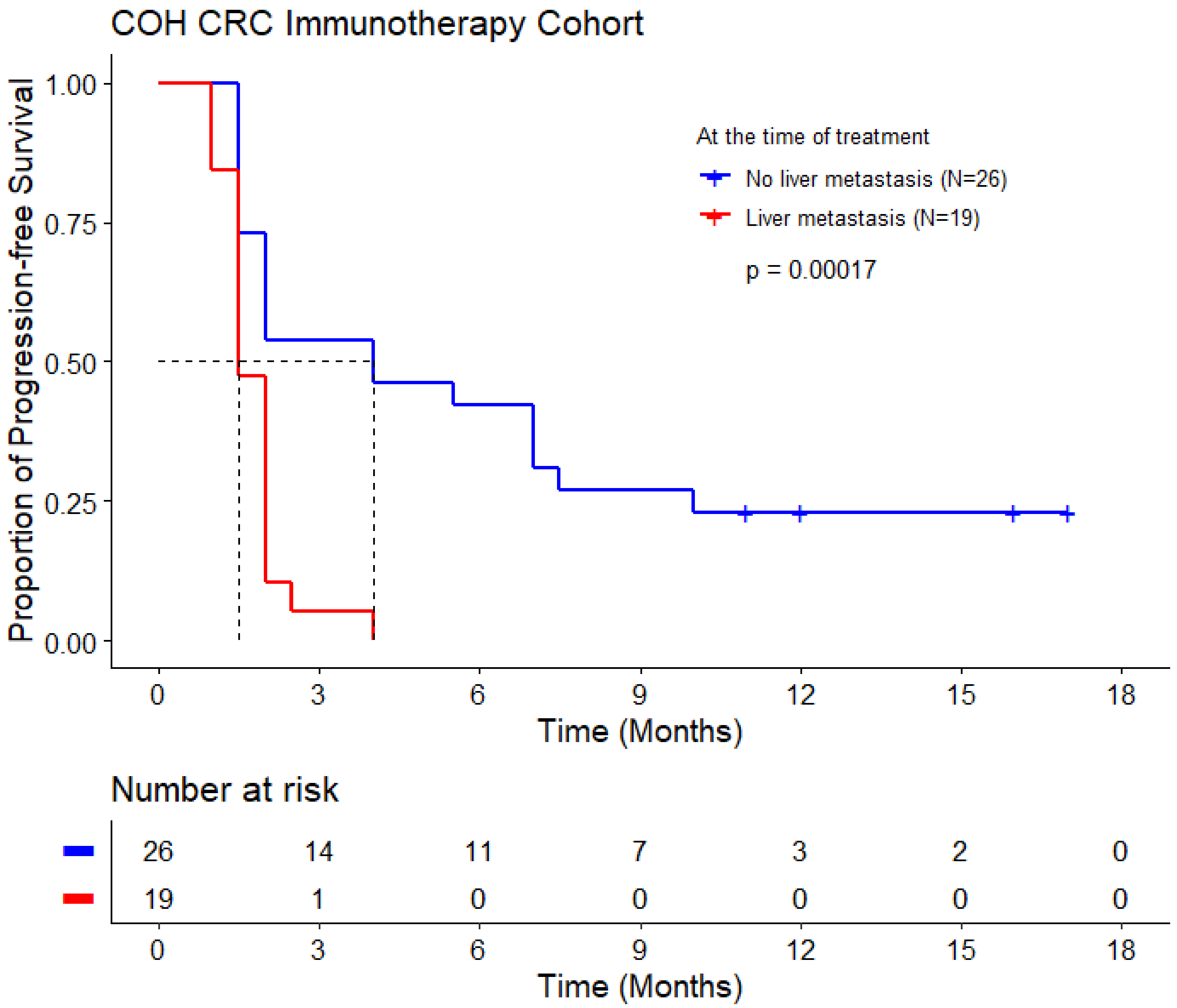

© 2021 Wang C et al. *JAMA Network Open*.
